# Outburst of a subglacial flood from the surface of the Greenland Ice Sheet

**DOI:** 10.1038/s41561-025-01746-9

**Published:** 2025-07-30

**Authors:** Jade S. Bowling, Malcolm McMillan, Amber A. Leeson, Stephen J. Livingstone, Andrew J. Sole, Felix S. L. Ng, Nanna B. Karlsson, Peter Nienow, Karla Boxall, Brice Noël, Michiel R. van den Broeke, Thomas Slater, Jennifer Maddalena, Louise Sandberg Sørensen, Sebastian B. Simonsen, Jérémie Mouginot, Romain Millan, Laura Melling, Liam Taylor, Angelika Humbert

**Affiliations:** 1 Lancaster Environment Centre, Lancaster University, Lancaster, UK; 2 UK Centre for Polar Observation and Modelling, Lancaster University, Lancaster, UK; 3 Centre of Excellence in Environmental Data Science, Lancaster University, Lancaster, UK; 4 Data Science Institute, Lancaster University, Lancaster, UK; 5 School of Geography and Planning, University of Sheffield, Sheffield, UK; 6 Department of Glaciology and Climate, Geological Survey of Denmark and Greenland, Copenhagen, Denmark; 7 School of Geosciences, University of Edinburgh, Edinburgh, UK; 8 Laboratory of Climatology, Department of Geography, SPHERES research unit, University of Liège, Liège, Belgium; 9 Institute for Marine and Atmospheric Research, Utrecht University, Utrecht, the Netherlands; 10 UK Centre for Polar Observation and Modelling, Department of Geography and Environmental Sciences, Northumbria University, Newcastle-upon-Tyne, UK; 11 Geodesy and Earth Observation, DTU Space, Technical University of Denmark, Kgs. Lyngby, Denmark; 12 Department of Earth System Science, University of California, Irvine, CA USA; 13 Institut des Géosciences de l’Environnement, Universite Grenoble Alpes, CNRS, Grenoble, France; 14 School of Geography, University of Leeds, Leeds, UK; 15 Alfred-Wegener-Institut Helmholtz-Zentrum für Polar- und Meeresforschung, Bremerhaven, Germany; 16 Department of Geosciences, University of Bremen, Bremen, Germany

**Keywords:** Cryospheric science, Hydrology

## Abstract

As Earth’s climate warms, surface melting of the Greenland Ice Sheet has intensified, increasing rates of sea-level rise. Observations and theory indicate that meltwater generated at the ice sheet surface can drain to its bed, where it flows relatively unhindered to the ocean. This understanding of water movement within and beneath ice sheets underpins the theoretical models that are used to make projections of ice sheet change. Here we present evidence of a destructive mode of meltwater drainage in Greenland. Using multiple satellite sources, we show that a 90-million-cubic-metre subglacial flood forced its way upwards from the bed, fracturing the ice sheet, and bursting through the surface. This phenomenon was triggered by the rapid drainage of a subglacial lake and occurred in a region where the ice bed was predicted to be frozen. The resulting flood caused a rapid deceleration of the downstream marine-terminating glacier. Our observations reveal a complex, bi-directional coupling between the ice sheet’s surface and basal hydrological systems and demonstrate that extreme hydrological forcing may occur in regions of predicted cold-based ice. Such processes can impact the ice sheet’s dynamics and structural integrity but are not currently considered in ice sheet models.

## Abstract (web summary)

Satellite observations over the Greenland Ice Sheet reveal a destructive mode of meltwater drainage whereby a subglacial flood induced by the rapid drainage of a subglacial lake burst through the surface, fracturing the ice sheet.

## Main

Over the past three decades, the Greenland Ice Sheet lost an average of ~169 billion tonnes of ice annually, contributing a total of ~14 mm to global sea-level rise^1,2^. Approximately one-half of this mass loss originated from surface mass balance processes^2,3^, driven primarily by enhanced melting and run-off from the ice surface. As Arctic warming continues^4^, the intensity and extent of Greenland surface melting are projected to increase^5^, leading to greater ice mass loss^6,7^, and increased liquid water atop, within and beneath the ice sheet.

Understanding the passage of meltwater from its origin to the ocean is critical for assessing Greenland’s future contribution to sea-level rise and its impact upon the wider Arctic system. It is well established that meltwater generated at the ice sheet surface penetrates to the bed via moulins and crevasses^8–10^. Observational studies, mainly in southwest Greenland, have shown that the subglacial hydrological system rapidly responds to water input and that temporal variability in subglacial water flow exerts a key control on the overlying ice dynamics^9,11–15^. As such, it is necessary to determine both the mode (continuous versus episodic) and pathways (surface, englacial or subglacial) of water drainage and the extent to which meltwater is stored during this journey^16,17^. These factors affect many processes, including ice dynamics, thermodynamics, ice–ocean interactions, fjord circulation, primary productivity and rates of sediment and nutrient transfer to the ocean^18^.

One of the most recently discovered, yet poorly understood, components of Greenland’s subglacial hydrological system is its network of active subglacial lakes^19–24^. These are primarily fed by surface meltwater penetrating to the ice sheet base^20–23,25^ and have the potential to force large volumes of water through the subglacial system when they drain, altering its morphology and also the dynamics of the surrounding ice^26–29^. Currently, however, detailed observations of Greenland subglacial lake drainage events are rare, and process understanding of their drivers and impacts is limited. Indeed, basal thermal conditions, a key control on subglacial water distribution and dynamics, still remain uncertain across a substantial proportion of the ice sheet^30^. Against a backdrop of enhanced run-off during the twenty-first century^31,32^ and expected increases in the frequency and extent of subglacial lake drainage^23,33^, the consequent impact of these poorly understood events upon the ice sheet remains uncertain. Here we present observations of a destructive mode of Greenland subglacial lake drainage and analyse its impact upon the surrounding ice sheet.

## Subglacial lake drainage in North Greenland

**Fig. 1:**
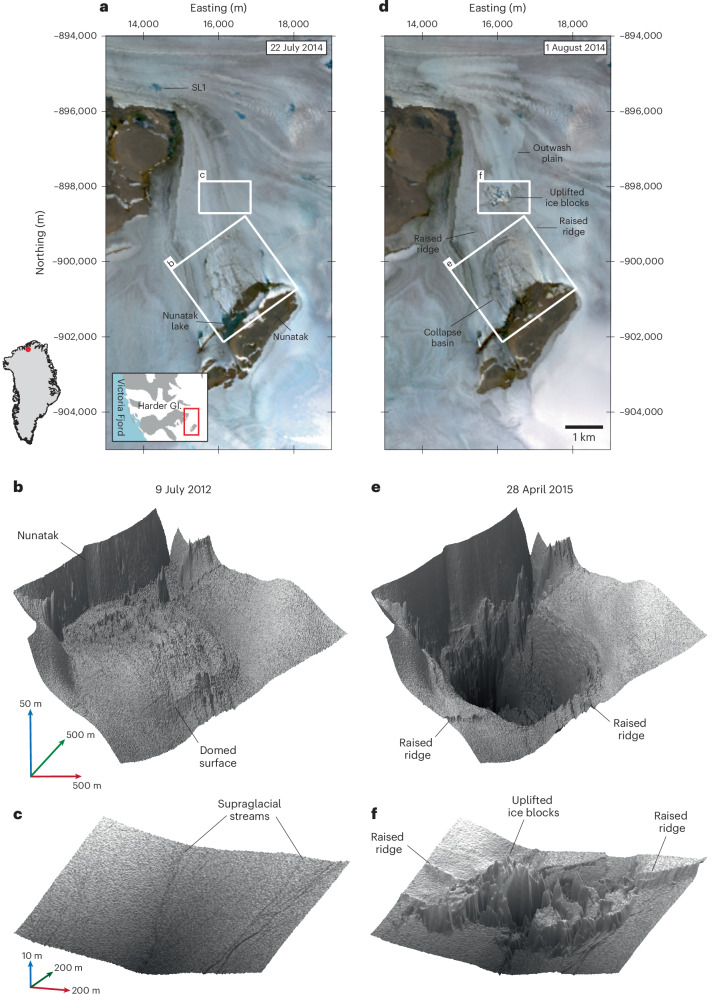
Observations of subglacial lake drainage and surface outburst. **a**, True colour composite Landsat-8 scene acquired on 22 July 2014 before the subglacial-outburst flood (SL1 indicates the location of a supraglacial lake referred to in the text; white boxes locate the regions shown in **b**, **c**, **e**, and **f**). Inset: the location of **a** (red box) relative to the Harder Glacier (Gl.) and Victoria Fjord. **b**, Three-dimensional shaded relief of the collapse basin mapped before lake drainage, using 2-m-resolution ArcticDEM data acquired on 9 July 2012. **c**, Three-dimensional shaded relief of the downstream region on 9 July 2012. **d**, True colour composite Landsat-8 scene acquired immediately after the subglacial lake drainage and surface outburst on 1 August 2014. **e**, Three-dimensional shaded relief of the collapse basin acquired on 28 April 2015, after the subglacial lake drained. **f**, Three-dimensional shaded relief of the same downstream region on 28 April 2015, showing ice fractures and uplifted ice blocks. Landsat-8 data in **a** and **d** from https://earthexplorer.usgs.gov and ArcticDEM data in **b**, **c**, **e** and **f** from https://data.pgc.umn.edu/elev/dem/setsm/ArcticDEM.

**Fig. 2:**
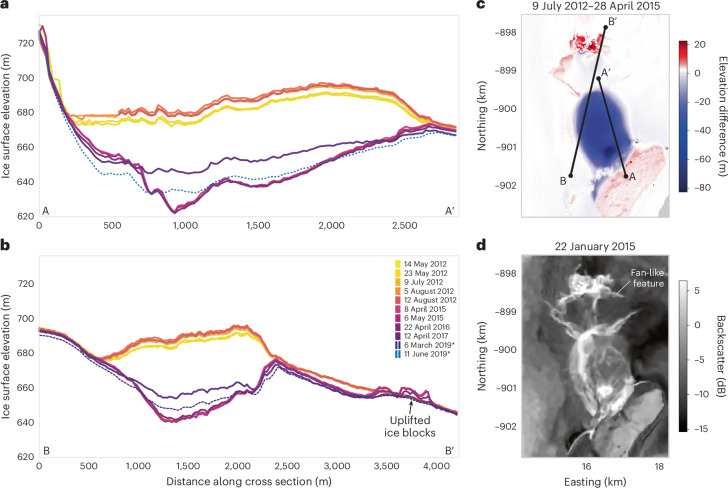
Changes in ice geometry and structure above the Harder subglacial lake. **a**, Repeat elevation profiles A–A′ (location shown in **c**) from sequences of co-registered ArcticDEM data (solid lines) and ICESat-2 data (dashed line) along ICESat-2 track 1130. **b**, Repeat elevation profiles B–B′ (location shown in **c**) along ICESat-2 track 1032, crossing both the lake and the edge of the downstream fracture site (entries marked by asterisks in the legend indicate data from ICESat-2). **c**, Surface elevation change between 9 July 2012 and 28 April 2015, from repeat ArcticDEM data. **d**, Sentinel-1 SAR backscatter image acquired after lake drainage (22 January 2015), showing evidence of fracturing of the ice surface. Data in **a** and **b** from https://data.pgc.umn.edu/elev/dem/setsm/ArcticDEM and https://nsidc.org, data in **c** from https://data.pgc.umn.edu/elev/dem/setsm/ArcticDEM, and data in **d** from https://browser.dataspace.copernicus.eu/.

**Extended Data Fig. 1:**
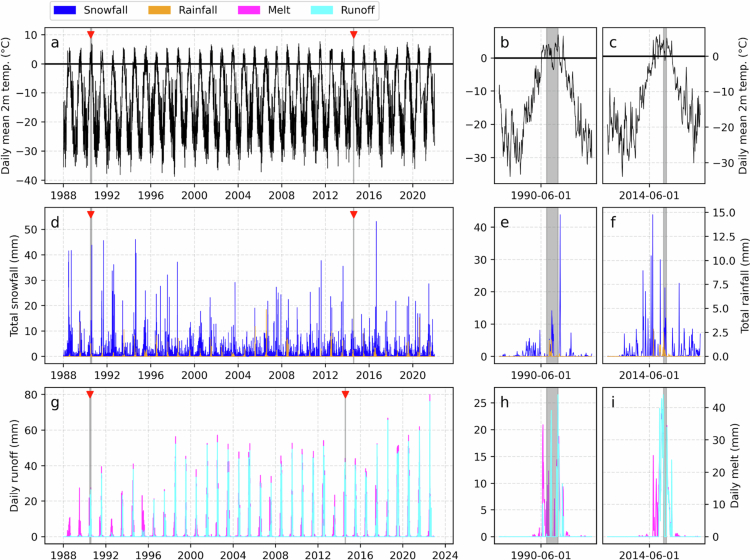
Background climatology derived from the statistically downscaled 1 km resolution RACMO2.3p2 at 81.70°N, 43.97°W, for the period 1988-2022. **a** 2 m temperature between 1988-2020. **b** Daily 2 m temperature for 1990. **c** Daily 2 m temperature for 2014. **d** Daily total snowfall (blue) and rainfall (orange) between 1988-2022. **e** Daily total snowfall and rainfall for 1990. **f** Daily total snowfall and rainfall for 2014. **g** Daily total runoff (turquoise) and melt (pink) between 1988-2022. **h** Daily total runoff and melt for 1990. **i** Daily total runoff and melt for 2014. The areas of grey shading, highlighted by the red arrows, mark the periods during which lake drainage occurred.

We use very high-resolution ArcticDEM digital surface models (DSMs)^34^ and satellite imagery to identify and monitor a previously undetected subglacial lake in northern Greenland, close to the marine-terminating Harder Glacier (81.5° N, 44.48° W) (ref. ^35^). During a 10-day period in the summer of 2014 (22 July–1 August), a 2 km^2^ region of the ice surface dropped by 85-m elevation, forming a deep collapse basin in the ice surface (Fig. 1). This rapid deflation, which occurred ~25 km inland, on slow-flowing ( < 10 m yr^*−*1^) ice adjacent to the main glacier, followed shortly after the seasonal run-off maximum (Extended Data Fig. 1). Before this event, the basin surface had been rising, to form a ~10–15 m high dome above the surrounding ice (Figs. 1 and 2). Following the 2014 surface collapse, uplift of the ice within the basin resumed, before it subsided by ~10 m between 2017 and 2019 (Fig. 2). We interpret this dynamic behaviour to be the surface expression of an active subglacial lake, similar to those observed previously in Greenland and Antarctica^20–23,29,36–38^.

**Extended Data Fig. 2:**
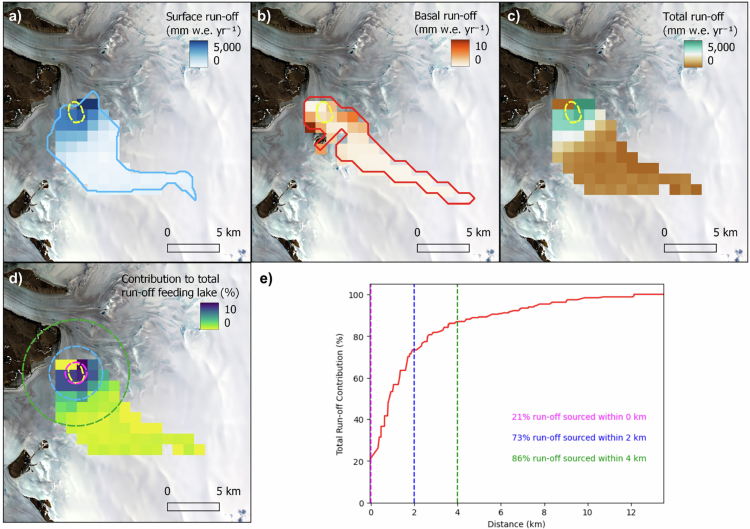
Meltwater sources feeding the Harder subglacial lake. (**a**) Mean annual surface run-off over the surface catchment feeding the lake, computed between 2010 and 2019, derived from the statistically downscaled 1 km resolution version of RACMO2.3p2; (**b**) Mean annual basal run-off over the basal catchment feeding the lake, computed between 2010-2019; (**c**) Total surface and basal mean annual run-off feeding the lake, computed between 2010-2019; (**d**) The spatial distribution of the proportion of total run-off feeding the lake, indicating that the majority of run-off was generated in close proximity to the lake; (**e**) The cumulative distribution of run-off feeding the lake, according to the distance travelled from source. The surface catchment is outlined in blue in panel a, the basal catchment is outlined in red in panel **b**, and the subglacial lake is outlined in dashed yellow in panels **a-d**. In panels d and e the pink, blue and green dashed lines indicate distances of approximately 0, 2 and 4 km from the subglacial lake. The background image in panels a-d is a true colour composite acquired by Landsat-8 on 22nd July 2014. Landsat-8 data in **a**–**d** from https://earthexplorer.usgs.gov.

Assuming that the volume of water discharged by the subglacial lake is approximated by the volume displaced by the newly created surface feature, we estimate that 9 × 10^7^ m^3^ of water drained from the lake during the 10-day period. Contemporaneously, a neighbouring supraglacial lake (abutting the adjacent nunatak) also drained, suggesting a direct connection between the surface and basal hydrological systems. Modelling of upstream surface and basal melting suggests that the subglacial lake was primarily fed from locally sourced surface meltwater, with more than 99% of water generated within its catchment originating from surface melt and 73% of all meltwater being generated within 2 km of the collapse basin itself (Extended Data Fig. 2). During the 10-day drainage window between satellite acquisitions, water drained from the lake at a mean rate of 100 m^3 ^s^*−*1^, with peak discharge probably much higher if drainage occurred over a shorter period. As such, the water flux was two orders of magnitude higher than that from an Antarctic lake of similar volume^33^ and one of the largest events recorded beneath the Greenland Ice Sheet to date^25,33^. Notably, the estimated discharge rate represented an ~ 30-fold increase in the daily meltwater run-off flux generated across the entire Harder Glacier catchment at that time, suggesting that lake drainage exerted a substantial perturbation on the downstream subglacial system.

## Surface outburst of the subglacial flood

**Extended Data Fig. 3:**
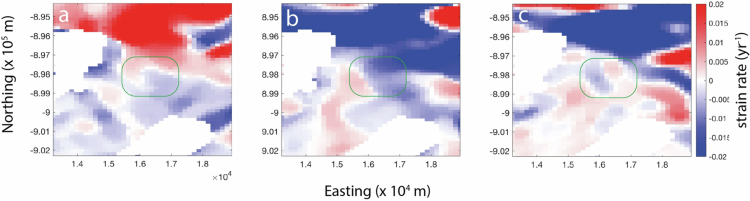
Strain rate components computed from the ice flow velocity fields. **a**. longitudinal, **b**. shear, and **c**. transverse strain rates, with each component smoothed using a sliding 5×5 pixel median filter. The green polygon indicates the location of the surface fractures, which coincides with where the longitudinal strain rate (panel **a**) switches from negative (compressional flow) to positive (extensional flow).

Theory and observations of lake drainage suggest that floodwater is routed subglacially, according to gradients in the hydraulic potential. As such, subglacial floods in Greenland form part of a broader unidirectional hydrological system, whereby surface meltwater is routed from the surface to the bed and, eventually, to the ocean. Here, however, we find evidence of a contrasting mode of ice sheet drainage pathway. Approximately 1 km downstream of the collapse basin, a newly formed zone of fractures appeared in the ice surface, consisting of crevassing and uprooted ice blocks with a combined height (crevasse depth plus ice block height) of 40 m (Figs. 1 and 2). Downslope of the fracture zone, an ~6-km^2^ region of the ice surface had been scoured clean. Together, these observations indicate that a substantial volume of water broke up through the ice at this location and flooded across the surface (Fig. 1). Indeed, the hydraulic potential difference between the lake and the fracture site indicates that the floodwater had sufficient hydraulic head to reach the surface at the fracture location. Notably, estimates of the longitudinal strain rate indicate a transition from compressional to extensional flow at the fracture site (Extended Data Fig. 3), which may have promoted fracturing through the ice column at this location.

**Extended Data Fig. 4:**
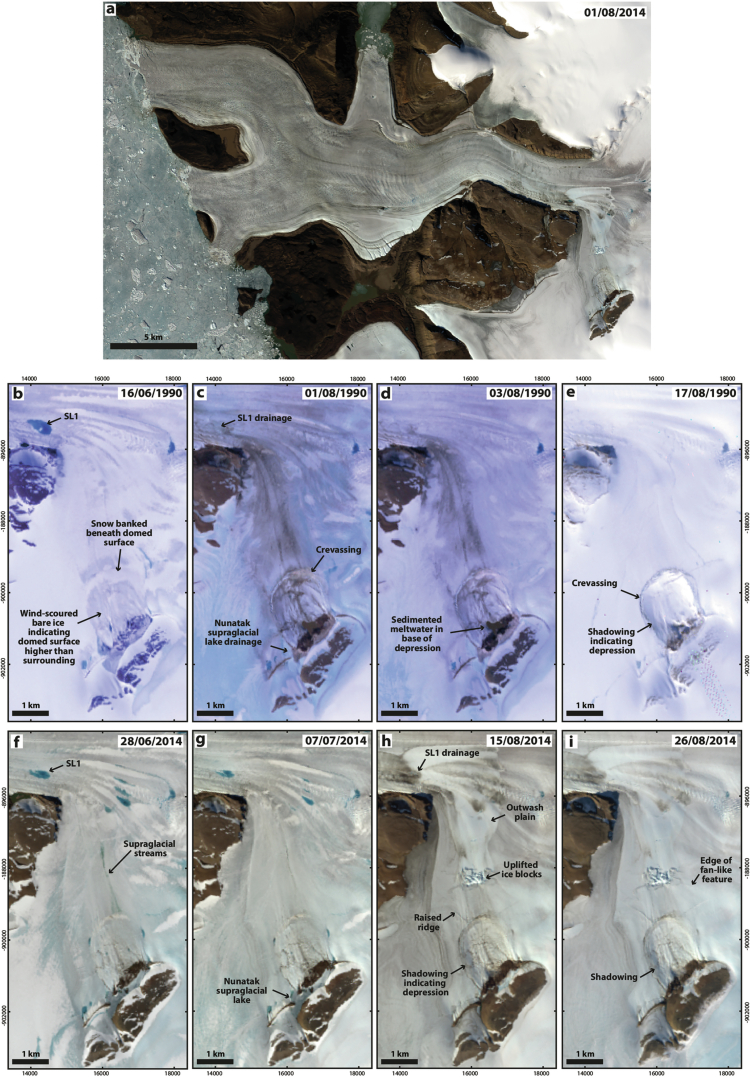
Evolution of the ice surface of the Harder Glacier following the two subglacial lake drainage events in 1990 and 2014. True colour Landsat 5 and 8 composites showing the evolution of the ice surface of the Harder Glacier following the two subglacial lake drainage events in 1990 and 2014. **a** Landsat scene showing the study area after the 2014 subglacial lake drainage event on 1^st^ August 2014 **b-e**. Time series of Landsat 5 images spanning the 1990 lake drainage event. **f-i**. Time series of Landsat 8 images spanning the 2014 lake drainage event. The 1990 lake drainage event was inferred from visual inspection of Landsat images dating back to 1985, to identify consecutive images that showed evidence of (1) a geometric change, from doming to a depression, in the surface, and (2) the formation of new fractures in the vicinity of the basin. Landsat 5 and 8 data from https://earthexplorer.usgs.gov.

Inspection of satellite imagery spanning 36 years shows that the distinctive oval-shaped surface feature above the subglacial lake has existed since at least 1985. Within this record, we find evidence for a single drainage event before 2014, which occurred between 21 June and 1 August 1990 (Extended Data Fig. 4) when the ice was more than 10 m thicker. In contrast to 2014, however, the 1990 drainage event did not drive downstream surface fracturing, implying that the floodwater ran entirely along the basal interface. Thus, we conclude that the 2014 surface outburst was unprecedented within the observational record.

## Downstream impacts of the 2014 subglacial lake outburst

**Extended Data Fig. 5:**
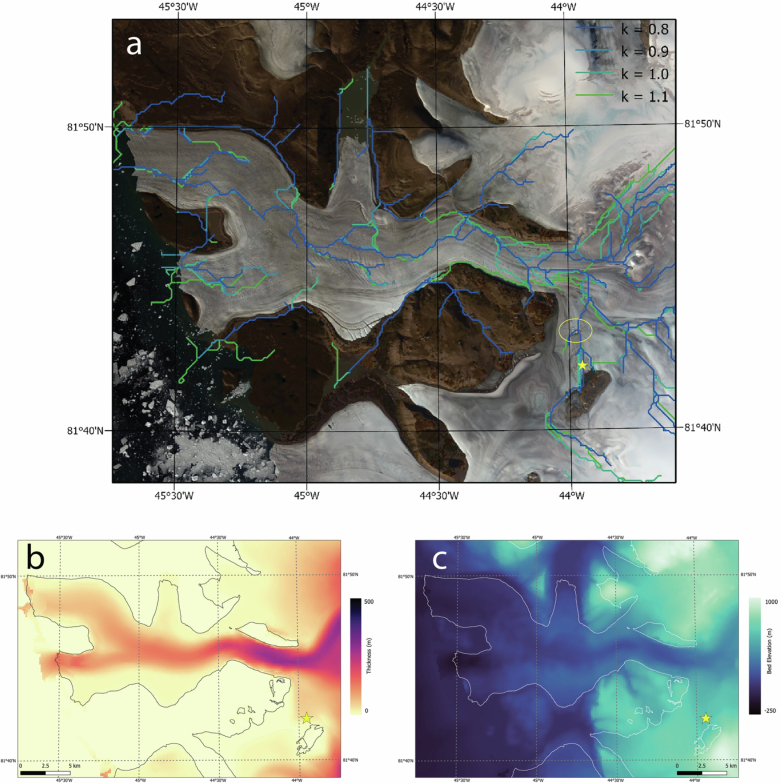
Ice thickness, bed elevation, and subglacial water routing pathways beneath the Harder Glacier. **a**. Subglacial water routing pathways beneath the Harder Glacier. The background image is a true colour composite Landsat-8 image acquired on 30^th^ July 2019. The green and blue coloured lines indicate modelled subglacial flow paths for a range of flotation factors, *k*, as indicated in the legend. The yellow star indicates the location of the Harder subglacial lake, and the yellow oval marks the location of the fracture zone. **b**. Ice thickness and **c**. bed elevation across the Harder Glacier region, determined from BedMachine version 3^46^. The yellow star indicates the location of the Harder subglacial lake and the black and white lines delineate the ice sheet margin, based upon the MEaSUREs Greenland Ice Mapping Project (GIMP) Land Ice and Ocean Classification Mask, Version 1 (ref. ^78^). Landsat-8 data in **a** from https://earthexplorer.usgs.gov.

**Extended Data Fig. 6:**
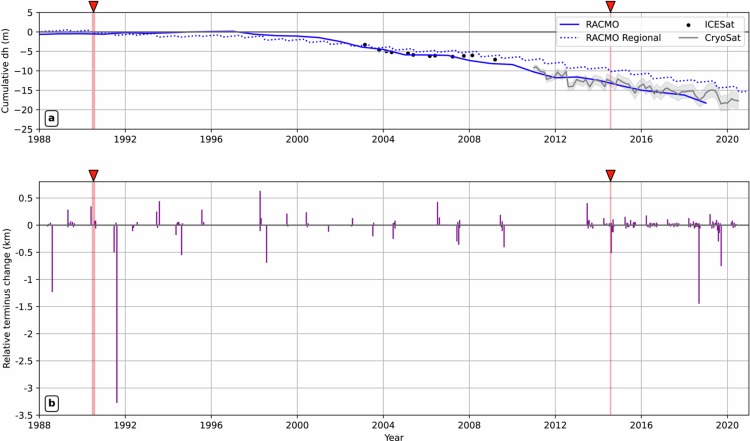
Change in ice elevation in the vicinity of the collapse basin and terminus position of the main northern lobe of the Harder Glacier. **a**. The long-term change in ice elevation in the vicinity of the collapse basin derived from CryoSat-2 (grey line; grey shading indicates the associated uncertainty of the elevation change) and ICESat elevation change (black points), and the 1 km resolution downscaled version of RACMO2.3p2^55^ at the location of the collapse basin (solid blue line) and a regional average (dashed blue line) spanning ice between 500 and 800 m a.s.l. **b**. Change in the terminus position of the main northern lobe of the Harder Glacier, measured using GEEDiT and MaQiT tools^77^; negative values indicate glacier front retreat, positive values indicate glacier advance. The vertical red lines mark the periods during which lake drainage occurred.

The area of flood-scoured ice ended abruptly after several kilometres, at a site where a supraglacial lake (denoted SL1 in Fig. 1a and Extended Data Fig. 4) also drained. The absence of surface scouring beyond this feature indicates that the floodwater, having burst upwards through the ice and flowed across the surface, then re-entered the englacial and subglacial system at this location, to flow onwards beneath the main trunk of the Harder Glacier. The synchronicity of the subglacial and surface lake drainage events suggests that the surface flood may have triggered the latter, most likely through a process of hydrofracture at the lake bed. Analysis of the basal hydropotential suggests that the floodwater was then routed beneath the Harder Glacier, to exit at the glacier’s main (northern) terminus (Extended Data Fig. 5). Within the same 10-day period, the terminus experienced a large calving event (500–600 m ice front retreat, the seventh largest event in the past 32 years; Extended Data Fig. 6). Whether there was a direct link to the lake outburst remains uncertain; however, these observations are consistent with a theory that a rapid pulse of water drove such a response, either by impacting the stress regime at the ice front, or by enhancing ocean-driven melting and undercutting^39,40^.

**Extended Data Fig. 7:**
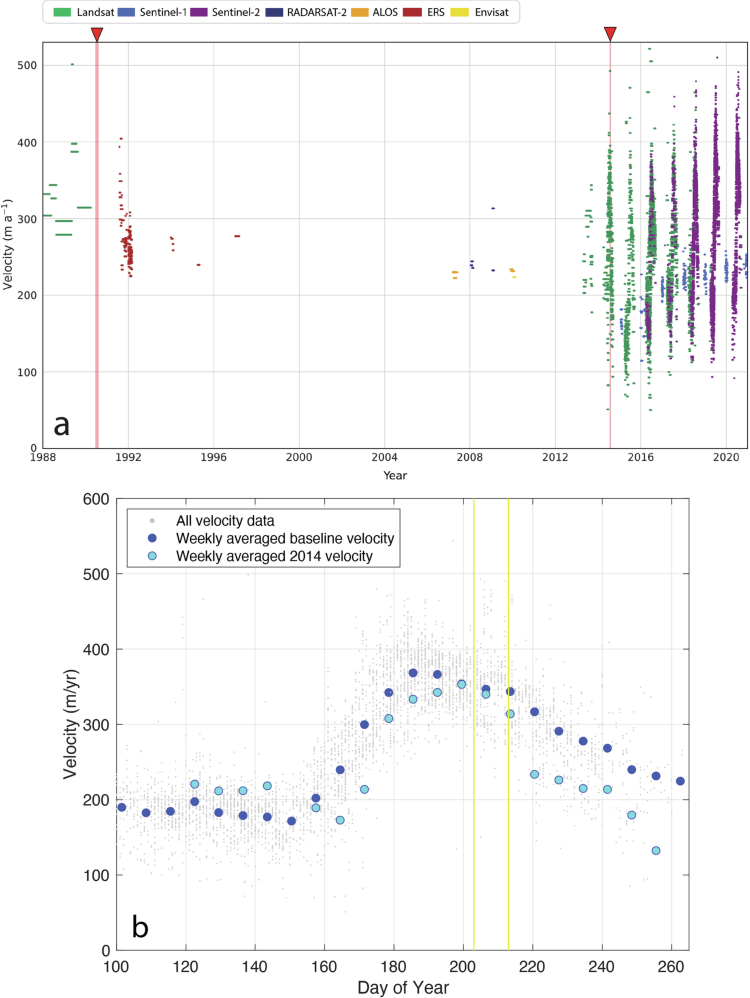
Velocity timeseries for the Harder Glacier. **a**. Velocity timeseries for the Harder Glacier between 1988 and 2020, for a location ~ 5 km inland of the ice front. The horizontal extent of each line represents the period between image pairs, and the vertical red lines indicate the timing of the 1990 and 2014 subglacial lake drainage events. **b**. Weekly-averaged seasonal evolution in velocity for all years excluding 2014 (blue) and for 2014 (turquoise), showing the unusually rapid seasonal deceleration in 2014 following the lake drainage. The yellow lines indicate the bounds of the lake drainage event, and the grey dots mark the individual velocities computed from all image pairs.

Our observations have shown that the lake drainage caused a substantial perturbation to the ice sheet’s hydrological system. To evaluate any associated impact upon downstream glacier dynamics, we computed 5,800 maps of ice velocity spanning 1985–2020 (Extended Data Fig. 7). These show that the dynamics of the Harder Glacier exhibit a pronounced seasonal cycle, superimposed upon a longer-term acceleration since 2014–2015. Whether lake drainage and the onset of acceleration in 2014 are causally linked (for example, due to a calving-induced reduction in buttressing) remains unclear, given the sparsity of data before 2013 and the numerous factors affecting marine-terminating glacier flow^41,42^. However, in the weeks following the outburst flood, there is evidence of a substantial shorter-term disturbance to the glacier’s dynamics, with a rapid deceleration of 80 m yr^−1^ (measured ~5 km inland from the ice front; Extended Data Fig. 7). Whereas late-summer deceleration is usual, in 2014 it was ~300% greater than normal, such that by 12 September, the glacier had lost 63% of its peak summer velocity, compared with an average reduction of 37% in other years. These observations are consistent with the theory that a rapid injection of floodwater increased the efficiency of the subglacial system, lowering basal water pressures and, ultimately, ice speed^43^. Such a mechanism is well established for supraglacial lake drainage and has been proposed for subglacial lake drainage beneath land-terminating ice^29^. Our observations suggest that it can impact fast-flowing, marine-terminating glaciers too.

## Thermal modelling and flood dynamics

Determining the broader implications of this destructive and poorly understood subglacial flood process requires an understanding of the physical characteristics of the system, including the basal conditions immediately downstream of the lake, which will have impacted the mechanism of flood initiation, propagation and outburst. Specifically, although our observations indicate that upwards-propagating hydrofracture occurred at the outburst location, it remains unclear whether subglacial lake drainage also initiated via hydrofracture along the basal interface (that is, basal hydrofracture^44^) or whether an initiation process associated with temperate ice masses (as inferred for exponentially growing *jökulhlaups*^45^) prevailed. In the latter case, ice-dam floatation or migration of a hydraulic divide may have occurred, with discharge then growing exponentially according to classical theory (widening of a pre-existing conduit due to frictional heating exceeding viscous closure). Although commonly invoked for lake drainage under temperate ice bodies, the second hypothesis assumes a thawed bed along the drainage pathway and does not explain the subsequent switch to upwards hydrofracture-driven propagation that is observed here. Conversely, although a hypothesis of basal hydrofracture is less established, its theoretical feasibility has been demonstrated^44^, and it places no dependency upon the presence of a temperate bed.

**Fig. 3:**
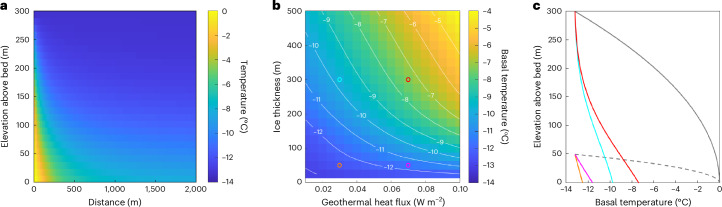
Simulations of thermal conditions downstream of the lake basin edge for different geometric and geothermal heat flux scenarios. **a**, Simulated temperature field, *T*(*y*, *z*), for a vertical section through the ice, for a high-end scenario (ice thickness = 300 m and geothermal heat flux = 0.07 W m^−2^). **b**, Basal temperature, *T*_b_, at the fracture site as a function of ice thickness and geothermal heat flux. The coloured circles correspond to the parameter choices for the experiments reported in **c**. **c**, Temperature profiles at the fracture site for four experiments exploring combinations of *H* = 50 m, 300 m and *G* = 0.03, 0.07 W m^−2^. The black solid and dashed curves plot the corresponding temperature boundary conditions at the edge of the lake basin, for *H* = 300 m and *H* = 50 m, respectively, which are defined as a parabolic vertical temperature profile to account for the proximity of the subglacial lake.

**Extended Data Fig. 8:**
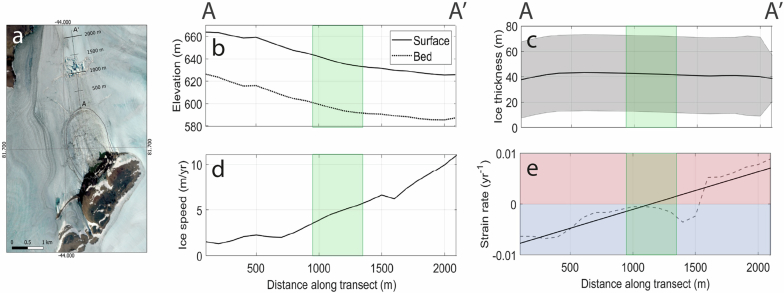
Ice geometry and dynamics immediately downstream of the collapse basin. Ice geometry and dynamics along a profile immediately downstream of the collapse basin, encompassing the region of surface fractures. **a**. the location of the profile, **b**. surface and bed elevations from BedMachine version 3^46^, **c**. ice thickness and associated uncertainty (grey shading) from BedMachine version 3^46^, **d**. ice velocity, and **e**. longitudinal strain rate. In panels **b-e** the green shading indicates the region where ice fracturing is observed at the surface. In panel e the blue and red shading indicate compressional and extensional flow, respectively, the dashed line denotes the longitudinal strain rate, and the solid line is a linear fit to the longitudinal strain rate, showing the large-scale trend across the region of interest. Data in **a** from https://browser.dataspace.copernicus.eu/.

To assess these two hypotheses, we modelled ice temperature along the slow-flowing ( < 10 m yr^−1^) section of the flood path between the surface depression and flood outburst site. These simulations were designed to better understand how climatic and geometric conditions (for example, surface temperature and ice thickness) might control the basal thermal state along this section. As ice thickness and geothermal heat flux are poorly constrained in this region, we assessed a wide range of plausible configurations^46,47^. In all cases, modelled basal temperatures remained below −5 °C, indicating that the climate and geometry favoured the presence of a frozen bed before lake drainage (Fig. 3). Indeed, based upon the available ice thickness information (Extended Data Figs. 5 and 8), it is likely that similar thermal conditions persisted until the thicker, faster-flowing ice of the Harder Glacier was reached further downstream. Thus, we infer that either (1) the lake was surrounded and sealed by largely cold-based ice (with basal hydrofracture required to initiate lake drainage, and the lake maintained by latent heat from locally sourced meltwater) or (2) an additional heat source sustained a temperate bed immediately downstream of the lake, despite the prevailing climatic and geometric conditions (thereby enabling a drainage initiation process associated with a classical *jökulhlaup* model^48^). Although persistent and widespread penetration of surface meltwater could provide this additional heat source, it represents an additional requirement to maintain the necessary basal conditions and does not explain the subsequent switch to upwards-propagating hydrofracture. In contrast, although it is unprecedented within an ice sheet setting, a purely hydrofracture-driven process provides a simpler and theoretically plausible^44^ mechanism, with analogies to the observed role of a frozen bed in driving floodwater to the surface of the John Evans Glacier in the Canadian Arctic^49^.

**Fig. 4:**
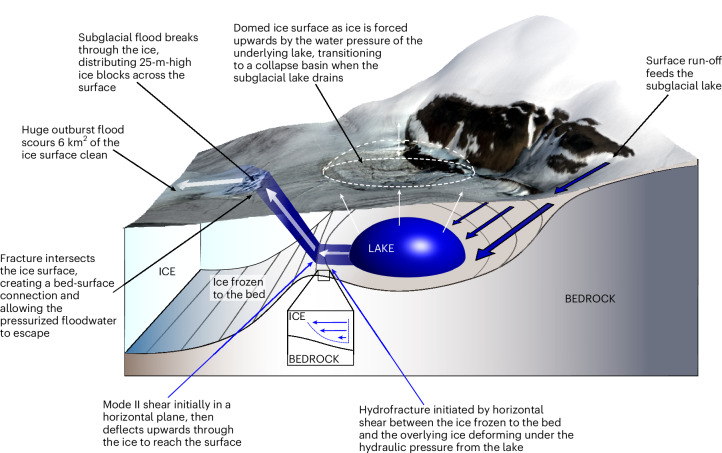
Conceptual model of the Harder subglacial lake drainage and surface outburst. The texts associated with blue arrows indicate tentative hypotheses. Note that the graphic is not to scale and the precise angle of flood propagation upwards to the surface remains unknown. Landsat-8 surface imagery data from https://earthexplorer.usgs.gov.

If correct, the hypothesis of basal and bed-to-surface hydrofracture represents a new type of ice sheet response to extreme hydrological forcing and poses the question as to why a crack initially propagating along the frozen basal interface subsequently deflected upwards to reach the surface. Whereas various stress regimes can drive hydrofracture through an ice column, it is unusual for surface fractures to be displaced from the source of the fracturing itself. For example, increasing tensile stresses induced by the upwards pressure of a filling lake would drive radial fractures above the lake itself. Such a pattern is not observed here. Instead, we propose a conceptual model of hydrofracture driven initially by increasing shear stresses along the ice-bed interface, as the force exerted outwards by the growing subglacial lake was resisted by ice frozen to the bed (Fig. 4). Such a regime has the capacity to drive in-plane (mode II) shear parallel to the bed and notably, under high stresses, to produce an upwards deflection in the angle of crack propagation^50^. Combined with the observed transition to extensional flow (Extended Data Figs. 3 and 8), which may have further encouraged ice fracturing at this location, this provides a tentative hypothesis as to why hydrofracture may have propagated upwards here. Conversely, the absence of surface fracturing during the 1990 drainage event suggests that the stresses may have been insufficient to propagate fractures in a secondary plane. Testing this conceptual model requires further dedicated observations and modelling to advance our process understanding of this event and, more broadly, the role of hydrofracture within an ice sheet setting.

This conceptual model of bed-to-surface hydrofracture through cold-based ice represents a mechanism that is currently absent from subglacial hydrological models, which have largely been developed using theory and observations relating to a warm basal interface^18^. Specifically, whereas existing work focuses on the dynamics and impacts of subglacial water flow in regions where there is high confidence that the bed is thawed, here we show that in regions where the bed is predicted to be frozen, or its thermal state remains uncertain, it is possible to subglacially store and rapidly release large volumes of locally sourced surface meltwater. Currently, an estimated 68% of Greenland’s bed has either a frozen (40%) or uncertain (28%) basal thermal state^30^. Furthermore, our observations suggest that in contrast to a classical model of lake drainage through temperate basal ice^48^, a more destructive process of hydrofracture may occur where the bed is frozen. As demonstrated here, this mechanism can damage the structural integrity of the ice and alter the dynamics of fast-flowing downstream glaciers. As Greenland’s climate undergoes future warming, surface melting will intensify and expand inland. Consequently, it is likely that meltwater will increasingly penetrate thin, cold-based ice at the margin and also access new areas of the bed that have previously exhibited a frozen or unknown thermal state. Within this context, our work highlights the need to fully understand the underlying theory of subglacial water flow and fracture mechanics in such systems and to better constrain the distribution and the dynamics of water beneath Greenland in its entirety.

We present evidence of a subglacial flood forcing its way upwards through the Greenland Ice Sheet in a region of predicted cold-based ice. This phenomenon was triggered by the rapid drainage of a subglacial lake, which was fed primarily by locally sourced surface meltwater. After initially flowing along the ice bed, the floodwater propagated upwards through the ice via hydrofracture, breaking through the surface and uprooting large ice blocks. Having flooded across the surface, the water re-entered the subglacial system, driving a substantial perturbation to the dynamics of the Harder Glacier. Such an outburst event is unprecedented in the observational record and demonstrates a high level of complexity and interconnectedness between the basal and surface hydrological systems of an ice sheet. These findings challenge the classical model that meltwater flow is always unidirectional from the ice sheet surface to its base, instead demonstrating that water can travel from the surface to the bed, and back again, over short spatial and temporal scales. Whether this phenomenon reflects an emerging response to increased surface meltwater penetration in regions of cold-based ice remains unclear. As such, our study highlights the need for better process understanding of the drivers and impacts of extreme perturbations to Greenland’s hydrological system.

## Methods

### Subglacial lake evolution

We used repeat measurements of surface elevation derived from timestamped ArcticDEM DSMs and from the Ice, Cloud and land Elevation Satellite-2 (ICESat-2) laser altimeter to study the evolution of the Harder subglacial lake. Two-metre-resolution ArcticDEM DSMs^34^ were generated by the ArcticDEM project, using stereoscopic WorldView and GeoEye satellite imagery^51^. We co-registered all the ArcticDEM stripfiles in the study area with seasonally aligned (March–May) ICESat-2 ATL06 data^52^, which was obtained from the National Snow and Ice Data Center for the period 2019–2020. Points scattered by cloud, blown snow or aerosols, or flagged as low quality, were discarded before analysis. Artefacts in the ArcticDEM stripfiles, together with dynamic surfaces such as the collapse basin, were masked before performing a least-squares planar fit to the residuals to model the vertical offset between the ICESat-2 and ArcticDEM elevations^53,54^:

1 $$\Delta z=a\,+{bx}+{cy}$$

Here, *x* and *y* are the horizontal coordinates, ∆*z* is the ArcticDEM–ICESat-2 elevation difference and *a*, *b* and *c* are the coefficients determined from the least-squares fit. We then removed the plane from the *z* component (elevation) of each DSM. A total of 1,352 ICESat-2 points were used for co-registering the 14 DSMs in the study area. To compute volume changes associated with the drainage of the subglacial lake, we first computed the area of the collapse basin, by differencing the pre-collapse and post-collapse ArcticDEM DSMs from 2014 and 2015, respectively. We then defined the extent of the collapse basin, based upon those pixels where the average elevation change between these dates was greater than the 1*σ* elevation variability^38^.

### Regional climate modelling

We used daily 2-m temperature, precipitation, melt and run-off from the 1-km downscaled version of the Regional Atmospheric Climate Model (RACMO) version 2.3p2 (RACMO2.3p2)^55^, which combines the dynamical core of the High-Resolution Limited Area Model (HIRLAM) and the physics of the European Centre for Medium-Range Weather Forecasts-Integrated Forecast (ECMWF-IFS cycle CY33r1). RACMO2.3p2 is forced by ERA5 reanalyses (1990–2020) and includes a 40-layer snow module that simulates melt, run-off, water percolation, retention and refreezing in firn. Snow layers were initialized using vertical temperature and density profiles from the Institute for Marine and Atmospheric Research Utrecht-Firn Densification Model. Full details are provided in Noël et al.^55,56^.

### Ice velocity and strain

We generated 5,800 maps of ice surface flow velocity between 1988 and 2020 by tracking the displacement of features in satellite optical^57,58^ (Landsat-8 and Sentinel-2) and Synthetic Aperture Radar (SAR)^59^ (Sentinel-1A/B, ENVISAT, RADARSAT-2, ALOS and ERS-1/2) imagery. To assess temporal changes in the Harder Glacier velocity, including anomalies in the 2014 seasonal evolution, we first extracted the average ice flow velocity within a 4 × 4 pixel box ~5 km inland from the terminus of the glacier (at 81.81° N, 45.40° W) for all image pairs and then we extracted weekly averaged time series. Finally, we computed the change in velocity in each calendar week, both before and after the 2014 drainage event, and also the trend in ice velocity over the six calendar weeks following the drainage event using linear regression.

To assess the strain regime in the vicinity of the outburst site, we calculated longitudinal, transverse and shear strain rates^60^:

2 $${\dot{\varepsilon }}_{\mathrm{lon}}={\dot{\varepsilon }}_{x}{\cos }^{2}\alpha +2{\dot{\varepsilon }}_{{xy}}\cos \alpha \sin \alpha +\,{\dot{\varepsilon }}_{y}{\sin }^{2}\alpha$$

3 $${\dot{\varepsilon }}_{\mathrm{trans}}={\dot{\varepsilon }}_{x}{\sin }^{2}\alpha +2{\dot{\varepsilon }}_{{xy}}\cos \alpha \sin \alpha +\,{\dot{\varepsilon }}_{y}{\cos }^{2}\alpha$$

4 $${\dot{\varepsilon }}_{\mathrm{shear}}=\left({\dot{\varepsilon }}_{y}-{\dot{\varepsilon }}_{x}\right)\cos \alpha \sin \alpha +\,{\dot{\varepsilon }}_{{xy}}(\,{\cos }^{2}\alpha -\,{\sin }^{2}\alpha )$$

where *α* is the flow angle defined counterclockwise from *x* axis (positive in the x direction) and ${\dot{\varepsilon }}_{x}$, ${\dot{\varepsilon }}_{y}$ and ${\dot{\varepsilon }}_{{xy}}$ are the components of the strain rate tensor defined according to Nye^61^:

5 $${\dot{\varepsilon }}_{x}=\frac{\partial u}{\partial x};{\dot{\varepsilon }}_{y}=\frac{\partial v}{\partial y};{\dot{\varepsilon }}_{{xy}}=\frac{1}{2}\left(\frac{\partial v}{\partial x}+\frac{\partial u}{\partial y}\right)$$

Here *u* and *v* are the two horizontal components of the observed surface flow velocity field. To perform this calculation in practice, we averaged surface flow velocity over the period 2013–2019 to maximize the signal-to-noise ratio and lower the uncertainties in ice flow direction compared with a single image-pair calculation^60^. Measuring any temporal change in the strain regime and also the strain regime at the time of the historical 1990 lake outburst was not possible, due to the precision of the feature tracking methods, the lack of coherence required for interferometry and the limited historical data availability.

### Thermal modelling

To assess the thermal conditions at the ice bed, we solve an energy equation for ice temperature, *T*(*y*, *z*), in the 2D glacier section between the collapse basin edge (*y* = 0 km) and the fracture zone (*y* ≈ 1 km), where *y* is the horizontal distance from the basin edge and *z* is height within the ice column:

6 $$v\frac{\partial T}{\partial y}+w\frac{\partial T}{\partial z}=\kappa \left(\frac{{\partial }^{2}T}{\partial {y}^{2}}+\frac{{\partial }^{2}T}{\partial {z}^{2}}\right)+\frac{{\tau }^{2}}{{\rho }_\mathrm{i}{c}_\mathrm{p}\eta }$$

Our model assumes steady state (∂*T* / ∂*t* = 0) as it is designed to simulate the long-term conditions before outburst and accounts for advective and conductive heat transport, together with viscous dissipation. In this model, *η* is the ice viscosity, $\tau$ is the effective stress and *v* and *w* approximate the velocity field. The parameters *κ*, *ρ*_i_ and *c*_p_ represent known ice properties, and we impose the surface temperature in 2014 as a boundary condition. The material constants used are the thermal diffusivity *κ* (36.1 m^2^ yr^−1^), density *ρ*_i_ (916 kg m^−3^) and specific heat capacity *c*_p_ (2 × 10^3 ^J kg^−1^ K^−1^) of ice. Given that ice velocity increases from ~ 0 m yr^−1^ at the basin edge to about 20 m yr^−1^ several kilometres downstream, both downward advection of cold ice and viscous dissipation are possible, and so our model accounts for both processes. Specifically, the flow configuration resembles flank flow off an ice divide, and so we specify the velocity field *v* ≈ *v*(*y*) = $y{\dot{\varepsilon }}_{{yy}}$ and *w* ≈ *w*(*z*) = $\mbox{-}z{\dot{\varepsilon }}_{{yy}}$ (which satisfies the continuity equation under plane flow) to capture the thermal advection to leading order. For the horizontal strain rate, we assume ${\dot{\varepsilon }}_{{yy}}=0.0035$ yr^−1^, which equates to the mean surface value over the first 1.5 km, as determined from our velocity observations. In the dissipation term of the model, the ice viscosity $\eta={[2\,A({T})\,{{\tau}^{n-1}}]}^{-1}$ is evaluated using Glen’s law exponent *n* = 3 and published^62^ values of the temperature-dependent factor *A*. The effective stress $\tau$ is calculated by Newton–Raphson iteration via ${\tau}^{2}={{\tau}_{yy}}^{2}+{{\tau_{yz}}^2}$ = (${\dot{\varepsilon }}_{{yy}}/A{\tau }^{n-1}$)^2^ + (*ρ*_i_ *g*(*H* – *z*)sin *α*_*s*_)^2^, where sin *α*_*s*_ = 0.02 is the ice surface slope and *g* = 9.81 m s^−2^ is gravitational acceleration.

Regarding the model boundary conditions, we specify *T* to be the surface temperature at *z* = *H*, ∂*T* / ∂*y* = 0 at *y* = 2 km and the geothermal heat flux condition −*k*_*i*_∂*T*/∂*z* = *G* at *z* = 0, where *k*_*i*_ = 2.1 W m^−1^ K^−1^ is the ice thermal conductivity and *G* is geothermal heat flux. The latter condition presumes a cold base with no sliding, which is consistent with our simulations that show *T*_b_ to be below the melting point. At the upstream boundary, *y* = 0, no information about the temperature in the ice column is available. In this regard, it is important to note that *y* = 0 locates the collapse basin edge and not necessarily the potentially dynamic margin of the subglacial lake; with lake water probably located at *y* < 0 while the lake was growing. As a conservative measure, we therefore account for the potential presence of relatively warm ice at the interface with the subglacial lake (which could serve as an additional source of heat to the downstream ice), by prescribing a parabolic temperature profile *T*(0, *z*) that decreases from the melting point at the bed to *T*_s_ at the surface. This boundary condition represents a stringent test for the existence of a cold bed further downstream because it prescribes the ice to be near the melting temperature for a considerable section of deep ice at the boundary (that is, it favours overestimation of *T*_b_). Internal model consistency is also established for alternative (less stringent) simulations where we replaced this boundary condition with ∂*T* / ∂*y* = 0 at *y* = 0 (based on the symmetry of an ice divide), which resulted in predicted *T*_b_ values that were uniformly more negative than those reported here.

Our simulations require knowledge of the ice thickness *H* and geothermal heat flux *G* at the study site, both of which are uncertain. Notably, *H* in the region of interest (within 1–2 km downstream of the collapse basin) has not been surveyed by in situ or geophysical methods. Although the proximity of a nunatak and bedrock to the northwest may suggest relatively thin ice, direct observational evidence is lacking. The ice thickness from BedMachine lies in the approximate range 30–50 m, with a nominal uncertainty of ~ ± 20 m. However, this uncertainty is itself uncertain, given the thickness is derived from bed topography interpolated by kriging from the nearby ice margin and the bed beneath the southern branch of Harder Glacier. *G* is also poorly constrained. A recent map of basal melt rates^63^ indicates *G* < 0.05 W m^−2^ for the catchments near Victoria Fjord, but our region lies just beyond the edge of its gridded data. Given these uncertainties, we perform multiple simulations across the parameter space 25 ≤ *H* ≤ 500 m and 0.01 ≤ *G* ≤ 0.1 W m^−2^ (bearing in mind that low *H* and low *G* in these ranges are most probable), to ensure the robustness of our conclusion of a frozen bed interface. For all simulations, we solve the energy equation by adding a relaxation time derivative to its left-hand side and evolving the temperature field to steady state, with *T*_s_ fixed at −13.2 °C (the mean surface temperature of the recent decade) and ignoring sub-annual temperature variations. This approach ensures conservative results, because accounting for (1) the reduction in heat retained in the ice column due to latent heat loss associated with any surface meltwater production during summer and (2) historically colder conditions (–14.6 °C in the 1960s) alongside contemporary glacier thinning (~13 m since 1990) within a time-dependent model would result in a colder ice column and, consequently, reduced *T*_b_. Finally, we test whether initially warm-based conditions beneath the surface outburst site could be self-sustainable under high geothermal flux supplemented by the heat released from basal sliding, by using a one-dimensional steady-state heat conduction model:

7 $$-{k}_\mathrm{i}{T}_\mathrm{s}/{H}_{\min }=G+(\;{\rho }_\mathrm{i}{\rm{g}}{H}_{\min }\sin {\alpha }_\mathrm{s}){v}_\mathrm{s}$$

where *H*_min_ is the minimum thickness for sustaining a warm base. The final term in the equation describes the heat dissipation from sliding. Because of its inclusion, this model directs the heat sources maximally to the basal interface; the model also ignores downward advective cooling and latent heat loss to basal melting, so it severely underestimates *H*_min_ to give a conservative test. Setting *T*_s_ = –13.2 °C (the higher of the decadal mean surface temperatures corresponding to the 1990 and 2014 drainage years), *G* = 0.1 W m^−2^ and *v*_s_ = 10 m yr^−1^ yields *H*_min_ = 243 m, which rules out a warm base between the lake and the 2014 outburst site unless the ice is much thicker than expected.

### Hydraulic potential

To investigate whether the conditions required to force water from the subglacial lake to the surface at the fracture zone were met, we assessed whether the lake hydraulic potential exceeded the hydraulic potential at the fracture site. If *z*_L_ and *z*_R_ denote the ice surface elevation above the lake (pre-outburst) and at the fracture zone, respectively, and *H*_L_ is the ice thickness above the lake, then this entails that *ρ*_i_ *gH*_L_ *+* *ρ*_w_*g*(*z*_L_ – *H*_L_) > *ρ*_w_*gz*_R_, where *ρ*_w_ = 1,000 kg m^−3^ is the density of water. Hence:

8 $${z}_\mathrm{L} - \frac{{\rho }_\mathrm{w}\,{{\mbox{-}}\; \rho }_\mathrm{i}}{{\rho }_\mathrm{w}}{H}_\mathrm{L} > {z}_\mathrm{R}$$

where *z*_L_ ≈ 690 m and *z*_R_ ≈ 655 m based upon the ArcticDEM surface elevation model. Equation (8) therefore constrains *H*_L_ to be less than 420 m. As the ice above the lake is probably thinner than this value, it is probable that this condition is met, in which case water from the lake can be forced to the surface at the fracture zone.

### Meltwater flux estimation and subglacial water routing

We modelled the surface and basal meltwater fluxes over both the subglacial lake’s and the Harder Glacier’s surface and basal upstream catchments, using estimates of (1) surface melt from the 1-km downscaled version of the RACMO2.3p2 (ref. ^55^) and (2) basal melt due to geothermal heat flux, frictional heating and the heat released by surface meltwater reaching the bed^63^. This analysis was designed to assess (1) the sources of water feeding the lake (via an assessment of the meltwater generated across the lake’s upstream catchment) and (2) the extent to which the drainage overloaded the Harder Glacier’s subglacial system (via an assessment of the melt generated over the entire Harder Glacier catchment itself). The derived run-off estimates represent an upper bound on the water available because they assume that (1) all surface water was routed to the bed (that is, no water is stored on top or within the ice) and (2) no refreezing occurred at the ice base. Where surface storage or basal refreezing does occur, this will reduce the background volume of water flowing through the subglacial system and hence the relative size of the perturbation caused by the lake drainage would have been even higher.

The routing of subglacial water was modelled using the Shreve hydraulic equation^64^:

9 $${{\varnothing }}={\rho }_{\mathrm{w}}g{z}_{\mathrm{b}}+k{\rho }_{\mathrm{i}}{gH}$$

where $\varnothing$ is the hydropotential, ${\rho }_\mathrm{w}$ and ${\rho }_\mathrm{i}$ are the densities of water and ice, respectively (1,000 and 916 kg m^−3^), ${z}_\mathrm{b}$ and *H* are the bed elevation and ice thickness^46^ and *k* is a ‘flotation factor’. We do not estimate routing at the time of the 1990 lake drainage because the spatially resolved ice thickness downstream of the lake site remains uncertain at that time. The flotation factor is the ratio between the subglacial water pressure and the ice overburden pressure, where *k* > 1.0 suggests the subglacial water pressure exceeds overburden and $k < 1.0$ suggests that subglacial water pressure is less than overburden. Because subglacial water pressure is unknown, we compute downstream routing pathways for the subglacial flood using a range of plausible *k* values (0.8, 0.9, 1.0 and 1.1) to account for this uncertainty^65^. Specifically, we calculate the hydropotential using equation (9), remove sinks, determine the number of upstream cells contributing to each cell (to identify flow paths) and retain all cells which are connected to more than 100 cells upstream (to remove small tributaries). For all four values of *k*, our analysis indicates that subglacial water flow from the subglacial lake basin is routed to the northern lobe of the Harder Glacier. To delineate the upstream catchments used to compute the surface and basal meltwater fluxes, the ArcticDEM 100-m mosaic^34^ was used to define the surface catchments, and the subglacial catchments were estimated by calculating the hydropotential (equation (9)) using the surface and bed topography from BedMachine v3^46^, assuming that the subglacial water was at overburden pressure, and then delineating the resulting drainage basin by following the steepest gradients in the hydropotential.

### Ice thickness change

Ice thickness change between the 1990 and 2014 drainage events was computed in the vicinity of the lake site, based upon ICESat (2003–2009) and CryoSat-2 (2010–2020) observations, together with RACMO2.3p2 (ref. ^55^) simulations (1990–2020). CryoSat-2 Baseline-D Level-2I Synthetic Aperture Radar Interferometric (SARIn) altimeter observations were used to compute height evolution time series at 60-day epochs between October 2010 and October 2020, using a model fit method^66,67^, which was applied on a 5 × 5 km grid. This processing included a backscatter correction^68^ and filtering using the following statistical thresholds: a minimum of 70 data points, a minimum time series length of 2 years, a maximum root mean square difference of 12 m, a maximum elevation rate magnitude of 10 m yr^*−*1^ and a maximum surface slope of 5°. We then computed time series of height evolution by averaging the gridded elevation anomalies within 60-day intervals for the northern sector of the ice sheet where the subglacial lake is located (specifically, between elevations of 500 and 800 m above sea level, according to ArcticDEM^69^). ICESat laser altimeter measurements were used to compute elevation changes between 2003 and 2009. We used release 34 of the GLAS/ICESat Level-2 GLAH12 product^70^ processed with a plane-fitting method^71^ to obtain estimates of the temporal evolution of the ice surface. These data were pre-processed to remove the intercampaign bias^72^ and the saturation biases provided in GLAH12. RACMO2.3p2 simulations were used to compute 1990–2020 height change as a result of surface mass balance processes.

### Nunatak supraglacial lake volume

To estimate the volume of water stored in the supraglacial lake adjacent to the subglacial lake immediately before the 2014 subglacial lake drainage, we manually delineated the lake boundary to determine its extent, using a Landsat-8 scene acquired on 22 July 2014. We then estimated lake depth using a radiative transfer approach^73^, applied to the green band, which is expected to provide an upper bound on the lake volume^74^:

10 $$z=[\mathrm{ln}({A_\mathrm{d}}{\rm{{-}}}R_\infty ){\rm{{-}}}\mathrm{ln}({R_\mathrm{w}}{\rm{{-}}}R_\infty )]/g$$

Here *z* is the lake depth, *A*_d_ is the underlying lake bed reflectance, *R*_∞_ is the reflectance from optically deep water, *R*_w_ is the reflectance measured by the satellite and *g* is the coefficient for spectral radiance loss in the water column. To estimate *A*_d_, we used the mean reflectance values from the lake bed, using an image acquired on 1 August 2014 immediately after the lake had drained. *R*_*∞*_ was assumed to be 0, which represents a conservative lower bound, and *g* was estimated as 2kd, where kd is the diffuse attenuation coefficient for downwelling light^75,76^. Finally, to compute lake volume, depth estimates were integrated spatially over the lake area.

### Calving front change

Terminus positions of the Harder Glacier were manually digitized from all Landsat and Sentinel-2 optical imagery acquired between 1988 and 2020, using the Google Earth Digitization Tool^77^. Images obscured by cloud, or where ice mélange made it difficult to identify the calving front, were excluded. Changes in the calving front location were estimated using the centreline method in the Margin change Quantification Tool^77^.

## Online content

Any methods, additional references, Nature Portfolio reporting summaries, source data, extended data, supplementary information, acknowledgements, peer review information; details of author contributions and competing interests; and statements of data and code availability are available at 10.1038/s41561-025-01746-9.

## Data Availability

The ArcticDEM data used in this study are available from the Polar Geospatial Center (https://data.pgc.umn.edu/elev/dem/setsm/ArcticDEM/). The ICESat and ICESat-2 data can be accessed via the National Snow and Ice Data Center (https://nsidc.org/data). Sentinel-1 and Sentinel-2 data can be accessed via the Copernicus Browser (https://browser.dataspace.copernicus.eu/). Landsat-8 data can be accessed via the USGS EarthExplorer (https://earthexplorer.usgs.gov/). CryoSat-2 data are disseminated by the European Space Agency (ftp://science-pds.cryosat.esa.int/). The ice velocity and regional climate model data used in this study can be requested from the corresponding author. The BedMachine data are available from the National Snow and Ice Data Center (https://nsidc.org).

## Code availability

Code associated with this study is available via Github at https://github.com/jadebowling1/Harder-glacier-subglacial-outburst.
